# *RP2-*Associated X-linked Retinopathy: Clinical Findings, Molecular Genetics, and Natural History

**DOI:** 10.1016/j.ophtha.2022.11.015

**Published:** 2023-04

**Authors:** Michalis Georgiou, Anthony G. Robson, Katarina Jovanovic, Thales A. C. de Guimarães, Naser Ali, Nikolas Pontikos, Sami H. Uwaydat, Omar A. Mahroo, Michael E. Cheetham, Andrew R. Webster, Alison J. Hardcastle, Michel Michaelides

**Affiliations:** 1 Moorfields Eye Hospital, London, United Kingdom; 2 UCL Institute of Ophthalmology, University College London, London, United Kingdom; 3 Jones Eye Institute, University of Arkansas for Medical Sciences, Little Rock, Arkansas

**Keywords:** Genetics, Genotyping, Inherited retinal diseases, Phenotyping, Retinitis Pigmentosa, Retinopathy, *RP2*

## Abstract

**Purpose:**

To review and describe in detail the clinical course, functional and anatomic characteristics of *RP2*-associated retinal degeneration.

**Design:**

Retrospective case series.

**Participants:**

Male participants with disease-causing variants in the *RP2* gene.

**Methods:**

Review of all case notes and results of molecular genetic testing, retinal imaging (fundus autofluorescence [FAF] imaging, OCT), and electrophysiology assessment.

**Main Outcome Measures:**

Molecular genetic testing, clinical findings including best-corrected visual acuity (BCVA), qualitative and quantitative retinal imaging analysis, and electrophysiology parameters.

**Results:**

Fifty-four molecularly confirmed patients were identified from 38 pedigrees. Twenty-eight disease-causing variants were identified, with 20 not previously clinically characterized. Fifty-three patients (98.1%) presented with retinitis pigmentosa. The mean age of onset (range ± standard deviation [SD]) was 9.6 years (1–57 ± 9.2 years). Forty-four patients (91.7%) had childhood-onset disease, with mean age of onset of 7.6 years. The most common first symptom was night blindness (68.8%). Mean BCVA (range ± SD) was 0.91 logarithm of the minimum angle of resolution (logMAR) (0–2.7 ± 0.80) and 0.94 logMAR (0–2.7 ± 0.78) for right and left eyes, respectively. On the basis of the World Health Organization visual impairment criteria, 18 patients (34%) had low vision. The majority (17/22) showed electroretinogram (ERG) evidence of a rod-cone dystrophy. Pattern ERG P50 was undetectable in all but 2 patients. A range of FAF findings was observed, from normal to advanced atrophy. There were no statistically significant differences between right and left eyes for ellipsoid zone width (EZW) and outer nuclear layer (ONL) thickness. The mean annual rate of EZW loss was 219 μm/year, and the mean annual decrease in ONL thickness was 4.93 μm/year. No patient with childhood-onset disease had an identifiable ellipsoid zone (EZ) after the age of 26 years at baseline or follow-up. Four patients had adulthood-onset disease and a less severe phenotype.

**Conclusions:**

This study details the clinical phenotype of *RP2* retinopathy in a large cohort. The majority presented with early-onset severe retinal degeneration, with early macular involvement and complete loss of the foveal photoreceptor layer by the third decade of life. Full-field ERGs revealed rod-cone dystrophy in the vast majority, but with generalized (peripheral) cone system involvement of widely varying severity in the first 2 decades of life.

**Financial Disclosure(s):**

Proprietary or commercial disclosure may be found after the references.

Retinitis pigmentosa (RP) is a heterogeneous group of inherited retinal conditions, both in terms of phenotype and genotype, with a prevalence of 1/3000 to 1/4000 in the general population.^1^ Retinitis pigmentosa can be inherited in an autosomal dominant, autosomal recessive, or X-linked pattern.^1^^,^^2^ X-linked retinitis pigmentosa (XLRP) cases account for 15% of male individuals with simplex disease.^3^ X-linked RP is a severe form of RP, with most affected male individuals presenting with early-onset vision loss (< 10 years of age), nyctalopia, nystagmus, severely abnormal or undetectable electroretinogram (ERG), and progression to legal blindness by the third to fourth decade.^4^, ^5^, ^6^ Patients with XLRP have symptomatic night blindness from early childhood and are often myopic. *RPGR* and *RP2* disease-causing variants are the most common causes of XLRP accounting for 80% to 90% of cases.^1^ The ongoing gene therapy clinical trials for *RPGR*-associated XLRP^7^ were preceded by multiple studies describing in depth characterization of disease natural history.^8^, ^9^, ^10^, ^11^, ^12^, ^13^, ^14^ In contrast, the current literature describing the *RP2* phenotype is limited.

*RP2* disease-causing variants are responsible for 5% to 20% of XLRP.^15^, ^16^, ^17^, ^18^, ^19^, ^20^ The reports comparing the severity of *RPGR* and *RP2* XLRP have been inconclusive as to which genotype is associated with worse prognosis.^2^^,^^5^^,^^6^^,^^21^^,^^22^ The genotype-phenotype correlations in *RP2*-associated XLRP are limited.^23^ Differential diagnosis of *RP2-* or *RPGR-*XLRP is challenging, because no ocular measurement is genotype-specific.^4^^,^^5^ A tapetal-like reflex can be observed both in patients and carriers with *RPGR*- and *RP2*-XLRP.^24^

*RP2* (MIM 312600) is located on Xp11.23 and has a structure similar to cofactor C, which is involved in β-tubulin folding.^19^ *RP2* encodes a GTPase-activating protein for the small GTPase ARL3 and has a role in trafficking lipidated proteins in the retina to the outer segment of photoreceptors.^25^^,^^26^ Using retinal pigment epithelium and 3-dimensional retinal organoids differentiated from patient-derived inducible pluripotent stem cells with an *RP2* premature stop variant, read-through drugs, and adeno-associated virus gene therapy rescued the cellular phenotype, supporting a clinical trial in patients.^27^^,^^28^ However, there is currently a lack of robust natural history data in genetically proven patients with *RP2-*associated retinopathy. These data are needed to provide better informed advice on prognosis and optimize design of clinical trials including identifying possible robust outcome measures and participant stratification.

The current study provides a detailed characterization of the clinical phenotype, molecular basis, and natural history of a large series of patients with *RP2* retinopathy.

## Methods

### Subject Identification and Assessment

Male patients harboring disease-causing variants in *RP2* were identified from Moorfields Eye Hospital (London, UK) and University of Arkansas Medical Science retinal genetics clinics. All patients included were previously informed and consented. This retrospective study adhered to the tenets of the Declaration of Helsinki and was approved by the local ethics committees.

### Molecular Diagnosis

The majority of patients were screened using a diagnostic targeted next-generation sequencing panel for retinal dystrophy. Others were ascertained via research-based whole exome sequencing or targeted Sanger sequencing of *RP2*. Variants are annotated according to the Reference Sequence NM_006915. All variants have a gnomAD frequency of 0 (gnomAD v2.1.1). Splice site variants were assessed using SpliceAI (https://spliceailookup.broadinstitute.org).

### Clinical Notes

Clinical data extracted included age of onset, visual acuity, slit-lamp biomicroscopy, and fundoscopy findings. Symptoms at presentation and complications were also recorded. All available data were reviewed, including the findings at the last available follow-up.

### Best-Corrected Visual Acuity and Clinical Severity Grading

The best-corrected visual acuity (BCVA) was assessed monocularly with Snellen chart and converted to logarithm of the minimum angle of resolution (logMAR) for statistical analysis. Jayasundera et al^20^ have described an approach to subdivide *RP2*-XLRP patients into mild, less severe, and severe. Patients with relatively late-onset severe macular dysfunction were considered less severe. Best-corrected visual acuity with different cutoffs for different age ranges was used as a subjective surrogate for macular function. We adopted and adapted the same clinical severity grading criteria into logMAR and applied it for the best seeing eye (Table S1, available at www.aaojournal.org).

In addition, BCVA of the best seeing eye was used to categorize patients into 1 of 4 groups based on the World Health Organization visual impairment criteria that defines a person with no or mild visual impairment when presenting visual acuity is < 0.48 logMAR, moderate impairment when visual acuity is 0.48 to 1 logMAR, severe if 1 to 1.3 logMAR, and blindness if greater than 1.3 logMAR (Table S1). Low vision corresponds to patients with moderate and severe impairment. Counting fingers vision was given a value of logMAR 1.98, hand motion was given a value of logMAR 2.28, light perception and no light perception were specified as logMAR 2.7 and 3, respectively.^29^ The BCVA classification criteria are summarized in Table S1.

### Electrophysiological Testing

Pattern electroretinogram (PERG) and full-field ERG testing were performed in 22 patients, incorporating the standards of the International Society for Clinical Electrophysiology of Vision.^30^^,^^31^ Pattern ERG P50 was used as an objective measure of macular function, and the full-field ERG was used to assess generalized retinal function of rod and cone systems. The ERG data were compared with a reference range from a group of healthy subjects (age range, 10–79 years).^32^^,^^33^ The amplitudes of the main full-field ERG components were plotted as a percentage of the age-matched lower limit of normal or as a difference from the age-matched upper peak time limit, including the dark-adapted (DA) 10 ERG a-wave and the light-adapted (LA) 3 single flash ERG b-wave and the LA 3 30Hz ERG. To address non-Gaussian distribution within the control group, the limits were defined as the lowest amplitude value in the control group minus 5% of the reference range (maximum minus minimum values) for amplitudes or plus 5% of the reference range for peak times.^34^^,^^35^

### Fundus Autofluorescence

Fundus autofluorescence (FAF) images were obtained using short-wavelength excitation (488 nm) and a scanning laser ophthalmoscope according to previously described methods.^36^ Images were reviewed by one grader (M.G.) and qualitatively graded.

### OCT

The majority of patients seen over the last 15 years had both OCT and FAF imaging. Horizontal scans acquired using the Heidelberg Spectralis OCT (Heidelberg Engineering) were chosen for quantifying the residual ellipsoid zone width (EZW) using the foveal reflex as a reference point. In addition, the device was switched to follow-up mode, so that the same scanning location was imaged at the follow-up visit as the baseline. This enabled comparable measurements to be made between the 2 visits for a given subject. In others, the analysis described by Tee et al^37^ was used to align locations for follow-up measurements of retinal thickness and the EZW (described in detail in Supplementary Methods, available at www.aaojournal.org). Vendor supplied Heidelberg Eye Explorer (Heyex) software version 1.6.1.0 was used for image analysis and quantification of EZW, using the caliper tool.^38^

### Statistical Analysis

Statistical analysis was carried out using SPSS Statistics for Windows (Version 22.0, IBM Corp.). Significance for all statistical tests was set at *P* < 0.05. The Shapiro–Wilk test was used to test for normality for all variables.

## Results

### Molecular Genetics

**Figure 1:**
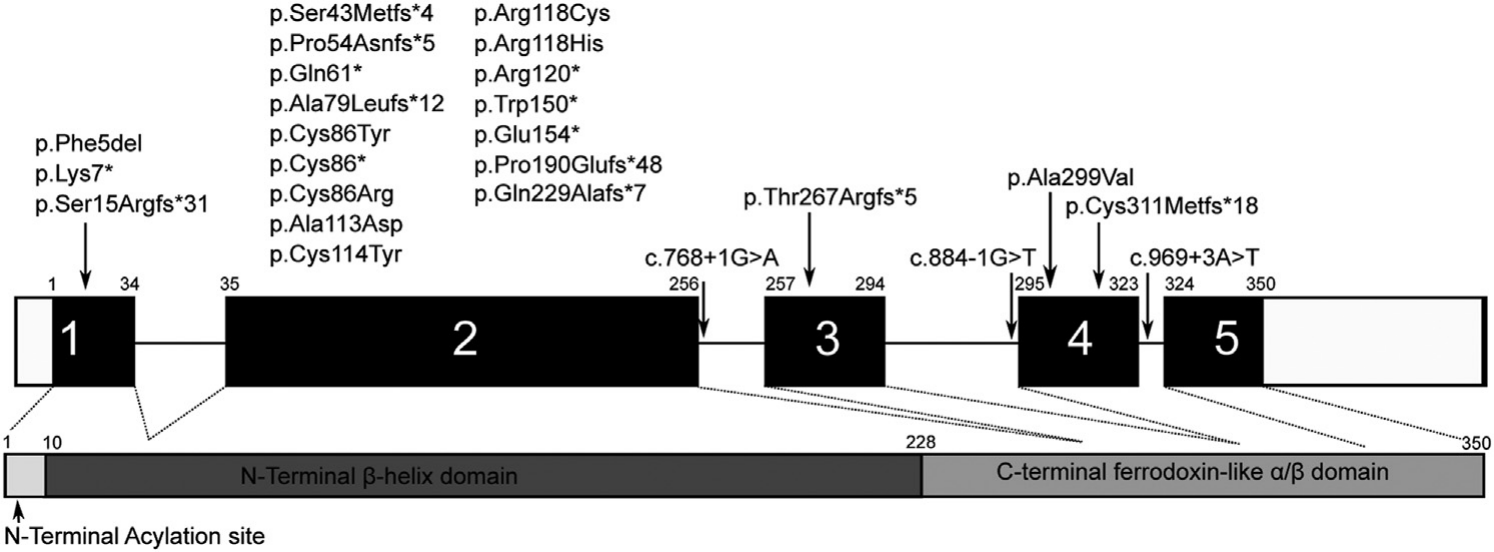
Schematic representation of variants in the RP2 gene and protein. The identified variants are marked along the corresponding location of the RP2 gene and protein. Black shaded boxes represent the coding exons (exons 1 to 5) separated by introns (solid line), with the protein domains (bottom) coded by each exon indicated with a dotted line.

A total of 54 molecularly confirmed patients were identified from 38 pedigrees. Twenty-eight variants were identified. The most common variant was c.358C>T p.(Arg120∗), identified in 5 pedigrees (13%); 6 variants were identified in 2 pedigrees each, and all others were restricted to single families. Identified variants included 8 frameshift alterations (28.6%), 7 missense (25.0%), 6 nonsense (21.4%), and 3 splice site changes (10.7%). One patient had a whole gene deletion, and 3 patients had smaller deletions. Twenty of the variants, including the deletions, have not been previously clinically characterized. Figure 1 shows the distribution of the variants across the *RP2* gene/protein. Table S2 (available at www.aaojournal.org) details the identified variants including their predicted effect.

### Phenotype, Age of Onset, and Presenting Symptoms

Fifty-three patients (98.1%) presented with RP. Age of onset was documented for 48 patients. The mean age of onset (range ± standard deviation [SD]) was 9.6 years (1–57 ± 9.2 years). Forty-four patients (91.7%) had childhood-onset disease (age range, 1–16 years), with mean age of disease onset (± SD) of 7.6 years (± 4.1 years). The 4 patients with adulthood-onset disease had mean age of onset 32.5 years (range, 17–57 years). One patient presented with symptoms and signs consistent with cone-rod dystrophy (1.9%) with onset of symptoms at age 10 years.

The first symptoms at disease onset were described in 48 of the patients with RP and included night blindness (n = 33, 68.8%), decreased central vision (n = 8, 16.7%), both night blindness and decreased central vision (n = 4, 8.3%), decreased central vision, and peripheral vision loss (n = 2, 4.2%). One patient with RP presented with nystagmus (n = 1, 2.1%). The patient with cone-rod dystrophy presented with decreased central vision and developed night vision difficulties later in life. Clinical data are summarized in Table 3.

**Table 3:** Clinical Data and Visual Impairment in *RP2* Rod-Cone Dystrophy

Parameter Characteristic	(n )	Mean (± SD Range)
**Age of Onset**		
Rod-Cone Dystrophy	48	9.63 ± 9.20, 1–57 yrs
Childhood onset	44 (91.7%)	7.55 ± 4.10, 1–16 yrs
Adulthood onset	4 (8.3%)	32.5 ± 16.15, 17–57 yrs
**Presenting Symptoms**∗		
Night blindness	33 (68.8%)	
Decreased central vision	8 (16.7%)	
Night blindness and decreased central vision	4 (8.3%)	
Decreased central and peripheral vision	2 (4.2%)	
Nystagmus	1 (2.1%)	
**BCVA**		
Age at baseline		23.2 ± 17.4, 3.8–71 yrs
Right eye mean BCVA at baseline		0.91 ± 0.80, 0–2.7 logMAR
Left eye BCVA at baseline		0.94 ± 0.78 0–2.7 logMAR
Mean follow-up	43	7.3 ± 7.1, 0.3–30.2 years
Right eye BCVA at follow-up		1.17 ± 0.84, 0.16–3.0 logMAR
Left eye BCVA at follow-up		1.16 ± 0.78, 0.16–3.0 logMAR
**Disease Severity**		
*Baseline*	53	
Mild disease	21 (39.6%)	
Severe disease	32 (60.4%)	
*Follow-up*	50	
Mild disease	10 (20%)	
Severe disease	40 (80%)	
**WHO Visual Impairment**		
No or mild visual impairment	24 (45%)	
Moderate impairment	11 (21%)	
Severe impairment	7 (13%)	
Blindness	11 (21%)	

BCVA = best-corrected visual acuity; SD = standard deviation; WHO = World Health Organization.

∗ The single patient with cone-rod dystrophy presented with decreased central vision.

### Genotype-Phenotype Correlations

Null and missense variants were present in childhood-onset and adulthood-onset groups. Of the 4 patients with adulthood-onset disease, 2 had frameshift variants with truncation/loss of function, 1 had a splice site variant with loss of donor splice site, and 1 had a substitution. In the childhood-onset group, the phenotype was uniform, with early-onset disease and early degeneration. No genotype-phenotype correlations were observed in the current report.

### Best-Corrected Visual Acuity

**Figure 2:**
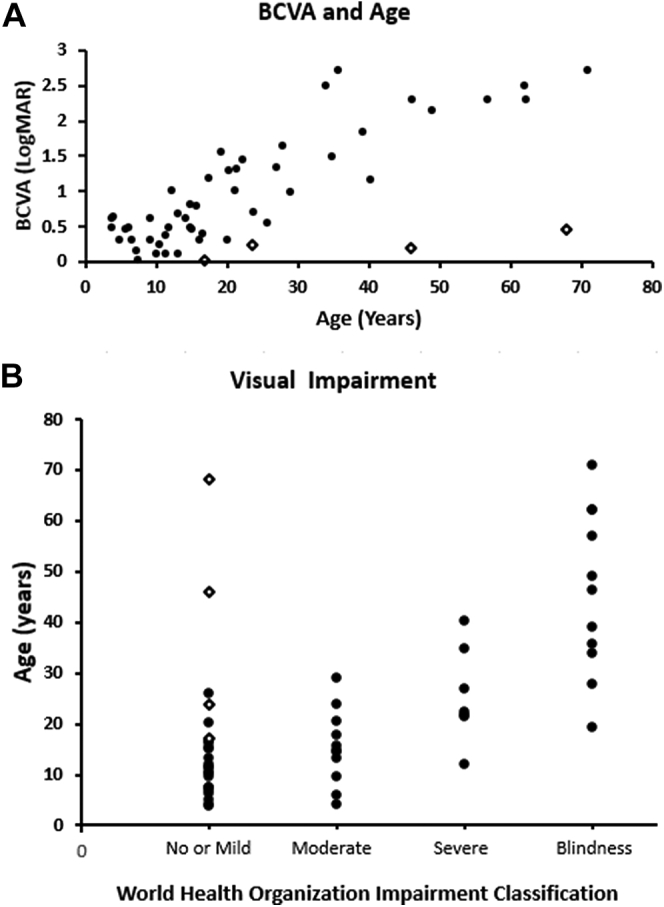
Visual impairment. **A**, Scattered plot graph presenting mean baseline best-corrected visual acuity (BCVA) against age. **B**, Stacked scatter plot depicts the age distribution among the different categories of visual impairment based on World Health Organization classification. As expected, a greater degree of impairment was present in older patients, with the exception of patients with adulthood-onset disease (open diamonds).

The BCVA was documented in at least 1 visit for 53 patients and was reduced in all cases. Mean age (range ± SD) for baseline BCVA for the whole cohort was 23.2 years (3.8-71 ± 17.4 years), with a mean BCVA (range ± SD) of 0.91 logMAR (0–2.7 ± 0.80) and 0.94 logMAR (0–2.7 ± 0.78) for right and left eyes, respectively. Forty-three patients had available longitudinal data, with a mean follow-up (range ± SD) of 7.3 years (0.3–30.2 ± 7.1 years). Mean BCVA change was 0.37 and 0.29 logMAR for the right and left eyes, respectively, for the follow-up period and was not statistically significantly different between right and left eyes (paired *t* test *P* < 0.05). Data for BCVA are summarized in Table 3 and mean baseline BCVA against age is presented in Figure 2A.

### Disease Severity

On the basis of previously described clinical severity grading criteria (Table S1), 21 patients had mild disease and 32 patients had severe disease at baseline. Of the 21 patients with mild disease, 18 were seen longitudinally. Eight of those 18 patients met the criteria for severe disease over a follow-up of 9.9 years (SD ± 4.8, range, 3–15.1 years). The 10 patients with mild disease at the last follow-up visit had significantly shorter follow-up time (mean ± SD, 4.8 ± 4.1 years), with 3 of them having later-onset adulthood disease.

According to the World Health Organization visual impairment criteria, 24 patients (45%) had no or mild visual impairment, 11 patients (21%) had moderate impairment, 7 patients (13%) had severe impairment, and 11 patients (21%) were blind. In total, 18 patients (34%) had low vision. Figure 2B depicts the age distribution for each class of visual impairment.

### Nonocular Manifestations

No nonocular manifestations were identified. However, ascertainment bias cannot be excluded, because the vast majority of patients were recruited from a stand-alone eye hospital (Moorfields Eye Hospital).

### Electrophysiology

There was a high degree of interocular ERG symmetry based on amplitudes of the DA 0.01, DA 3, and DA 10 ERG a- and b-waves, LA 30Hz ERG and LA 3 (single flash) ERG b-waves (slope = 0.94; *r*^*2*^ = 0.95), and the peak times of the DA 10 ERG b-waves and LA 30Hz ERGs (slope = 1.1; *r*^*2*^ = 0.86).

**Figure 3:**
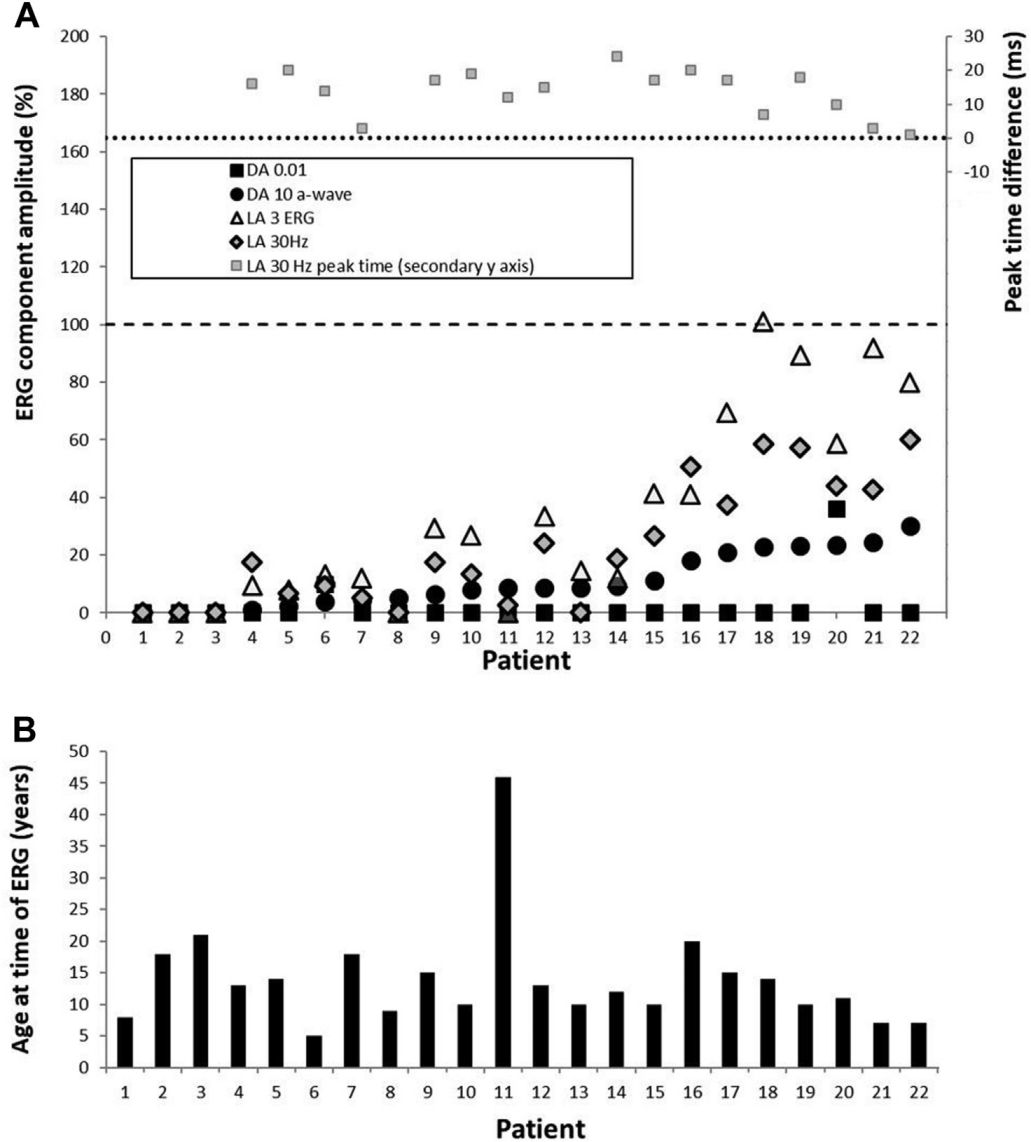
Full-field electroretinogram (ERG) findings summarized in 22 subjects tested according to the International Society for Clinical Electrophysiology of Vision standard methods. **A**, The amplitudes of the dark-adapted (DA) 0.01 ERG, DA 10 ERG a-wave, light-adapted (LA) 30 Hz ERG, and LA 3 ERG b-wave are plotted against the primary axis as a percentage of the age-matched lower limit of the (“normal”) reference range (horizontal broken line), with values arranged in ascending order of DA10 ERG a-wave amplitude for clarity. The LA 30 Hz peak times are plotted as a difference from the age-matched upper limit of normal timing (horizontal dotted line) against the secondary axis. **B**, The age of the patients at the time of testing, arranged in same order as in **A**.

**Figure 4:**
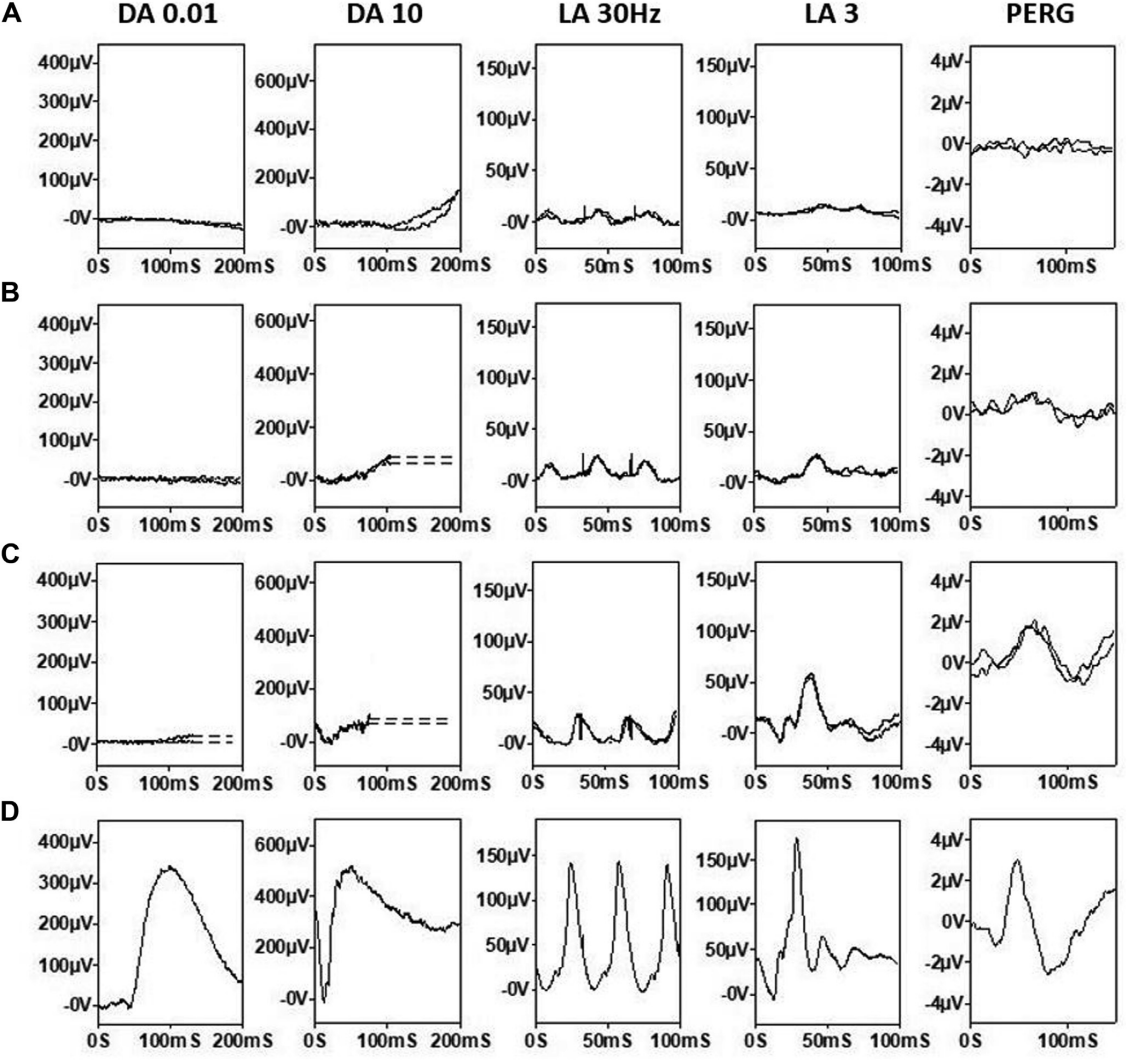
Representative full-field and pattern electroretinogram (ERGs). Patient 4 (**A**, aged 13 years), 12 (**B**, 13 years) and 21 (**C**, 7 years) correspond to the patient numbering used in Figure 3. Representative control (“normal”) recordings are shown for comparison (**D**). Data are shown for the right eyes only, because all showed a high degree of interocular symmetry. Patient traces are superimposed to demonstrate reproducibility. Broken lines replace blink artefacts for clarity. In all 3 patients, there is ERG evidence of rod-cone dystrophy. Pattern ERG P50 abnormalities are consistent with macular involvement: severe, moderate, or relatively mild. DA = dark-adapted; LA = light-adapted.

Three of 22 patients had undetectable full-field ERGs under all stimulus conditions (ages 8, 18, and 21 years), and 2 patients showed severe and similar reductions of DA and LA ERGs, consistent with a severe rod and cone photoreceptor dystrophy. The majority (n = 17), including the 11 with the mildest DA10 ERG a-wave reductions, showed better preservation of LA ERGs than DA 10 ERG a-waves, in keeping with a rod-cone dystrophy (Fig 3). All 17 patients with a detectable response showed delay in the LA 30Hz ERG, including the majority (n = 13) with severe delays of between 10 and 24 ms. Pattern ERG P50 was undetectable in all but 2 patients, including patient 12 (P50 delayed by 7 ms and reduced by > 70%; Fig 4B) and patient 21 (P50 delayed by 10 ms and reduced by > 25%; Fig 4C). Figure 3 summarizes the electrophysiological findings and patient ages at the time of testing, and Figure 4 shows representative recordings.

There was no significant correlation between age and the amplitudes of the DA 0.01 ERG, DA 10 ERG a- and b-waves, LA 30Hz ERG or LA3 ERG b-waves (maximum *r*^*2*^ = 0.083), or the peak times of the LA 30Hz (*r*^*2*^ = 0.025), although the narrow age range is highlighted (all but 1 patient were aged 5 to 21 years). Serial data were available in 1 child from the age of 7 years and revealed progressive PERG P50 reduction over 5 years and marked worsening of the DA 10 ERG between the ages of 10 and 12 years (Fig S5, available at www.aaojournal.org).

### Fundus Autofluorescence

**Figure 6:**
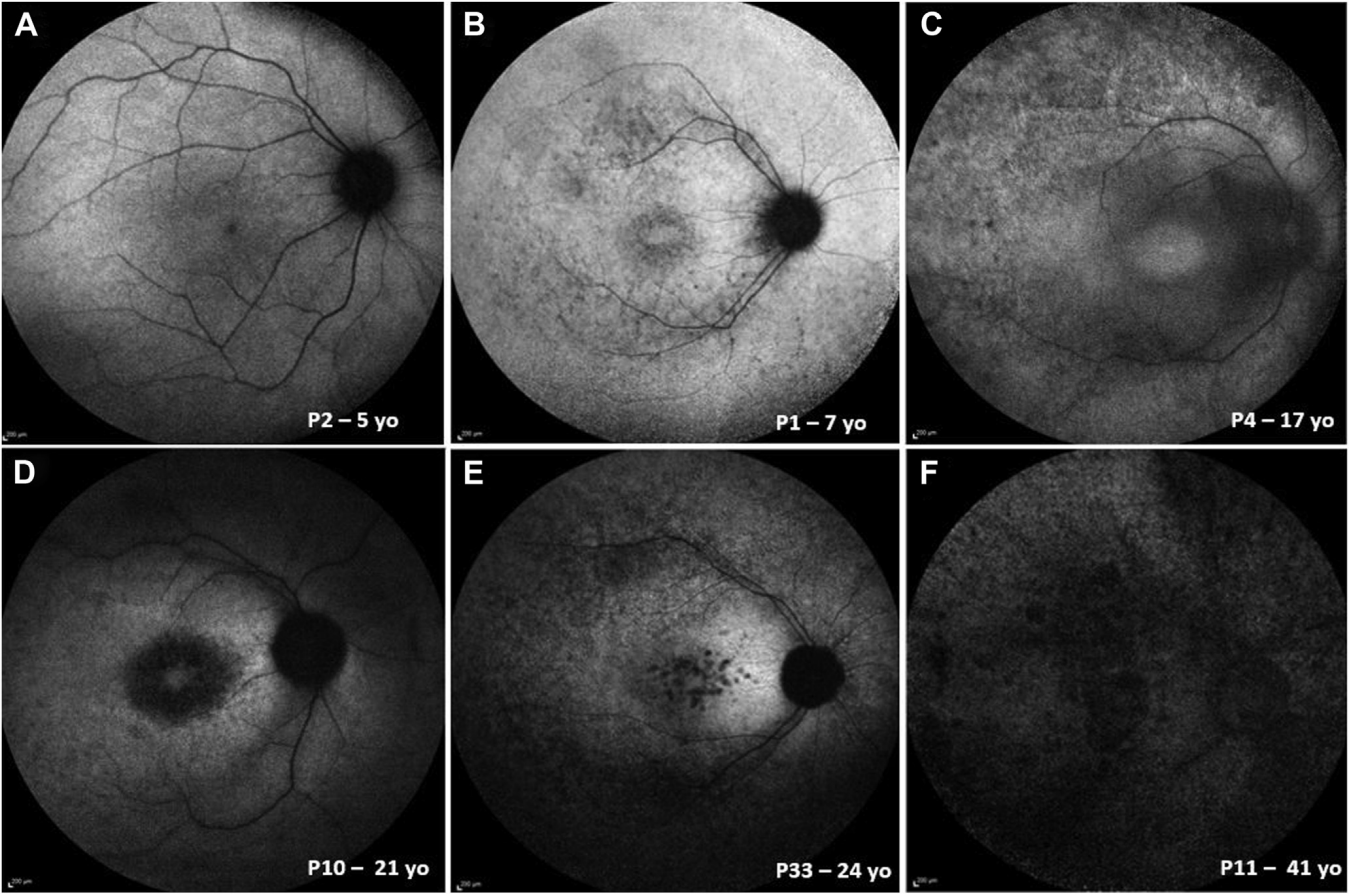
Fundus autofluorescence (FAF) imaging of 6 patients with RP2-associated retinopathy at different stages of the disease. **A**, Normal pattern of autofluorescence. **B**, Midperipheral patchy signal, with early patchy foveal pattern. **C**, Midperipheral patchy signal, with increased foveal signal. **D**, Atrophy, without midperipheral changes. **E**, Midperipheral patchy signal, with foveal atrophy. **F**, Diffuse atrophic changes.

Fundus autofluorescence imaging was available for 46 patients for at least 1 visit. At first evaluation, the mean age (± SD, range) was 25.1 years (± 16.7, 5.8–69.2 years). A range of FAF findings was observed, from normal FAF to advanced atrophy. Figure 6 shows examples of the different patterns of FAF observed. On qualitative assessment, we identified normal FAF in 11 patients (23.9%, mean age ± SD, range: 15.2 ± 11.1, 5.8–46.2 years). Two patients, aged 11 and 24 years, had a paracentral macular ring of increased signal; 6 patients (13%) had a macular ring of increased signal and midperipheral patchy changes with a mean age (± SD, range) of 18.71 (5.8, 11–25.9) years; 4 patients had patchy macular signal and midperipheral changes (8.7%, mean age ± SD, range: 26.0 ± 28.1, 6.7–68.2 years); a further 4 patients had normal macular signal with patchy midperipheral changes (8.7%, mean age ± SD, range: 27.4 ± 23.4, 12.2–62.1 years), and 1 patient had patchy macular signal with normal periphery. Eighteen patients (39.1%) had atrophy at a mean age of 34.0 years (± 15.1, 15.8–69.2). Three of the patients with advanced atrophy had a choroideremia-like pattern. Atrophy was the most common pattern on FAF imaging, and 31 patients (67.4%) had visible changes at the macula at baseline.

Follow-up FAF was available for 36 patients. The mean (± SD range) follow-up time was 6.0 years (± 4.3, 0.6–17.6 years). Nine of 9 patients with normal FAF at baseline showed abnormal changes (mean follow-up period of 6.4 years). The FAF showed a high degree of interocular symmetry in all cases, including those who had repeat imaging (examples shown in Fig S7, available at www.aaojournal.org**)**.

### OCT

Forty-six patients had at least 1 OCT imaging session. Baseline age (± SD, range) was 27.2 years (± 17.5, 5.2–69.2 years). The EZW and outer nuclear layer (ONL) thickness were not statistically significantly different between right and left eyes (paired *t* test *P* < 0.05). For further assessment, the mean EZW and ONL thickness for both eyes was calculated for each patient at each visit.

**Figure 8:**
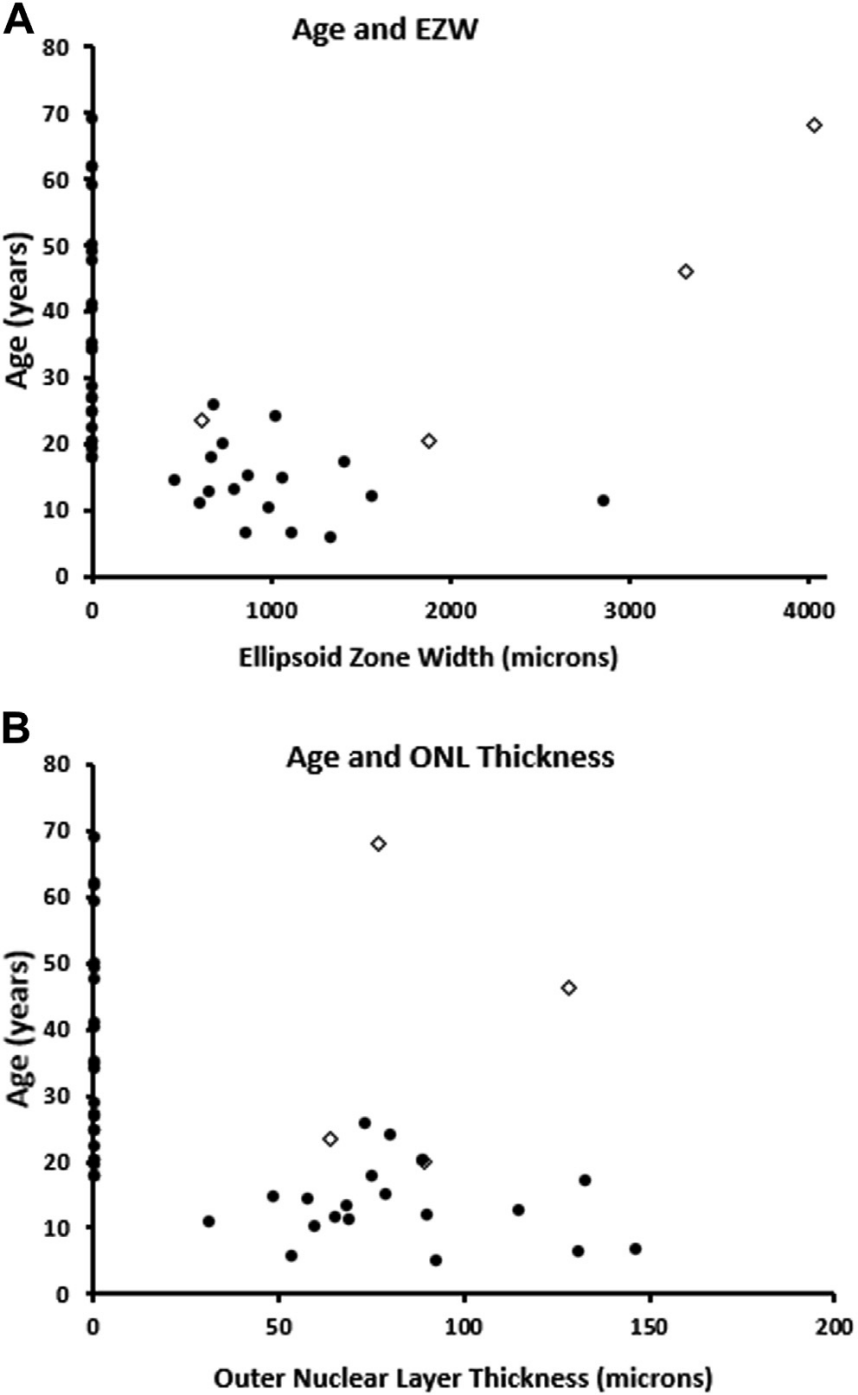
OCT graphs. Scattered plots presenting (**A**) ellipsoid zone width (EZW) and age, and (**B**) outer nuclear layer (ONL) and age. Greater degree of impairment of structural loss is present in older patients, except for patients with adulthood-onset disease (open diamonds). No patient with childhood-onset disease had identifiable ellipsoid zone (EZ) or ONL after the third decade of life.

Forty-two of the 46 patients had childhood-onset disease. Twenty-three patients had no identifiable ellipsoid zone (EZ) and complete ONL thickness loss at mean age (± SD, range) of 36.4 years (± 16.0, 17.9–69.2). Nineteen patients (mean age ± SD, range: 13.5 ± 5.7, 5.3–25.9 years) had identifiable EZ and residual ONL thickness. Mean EZW was 1493 (± 1496, 458–6280 μm), and mean ONL was 82 μm (31, 31–147 μm). Figure 8 presents the distribution of EZW and ONL thickness with age. Sixteen of the patients with identifiable EZ and ONL had longitudinal assessment, with a mean follow-up of 5.3 years. The mean annual rate of EZW loss was 219 μm/year, and the mean annual decrease in ONL thickness was 4.93 μm/year. No patient with childhood-onset disease had identifiable EZ after the age of 26 years at baseline or follow-up. Figure S9 (available at www.aaojournal.org) shows representative examples of OCT scans from 3 adult patients with complete EZ loss.

**Figure 10:**
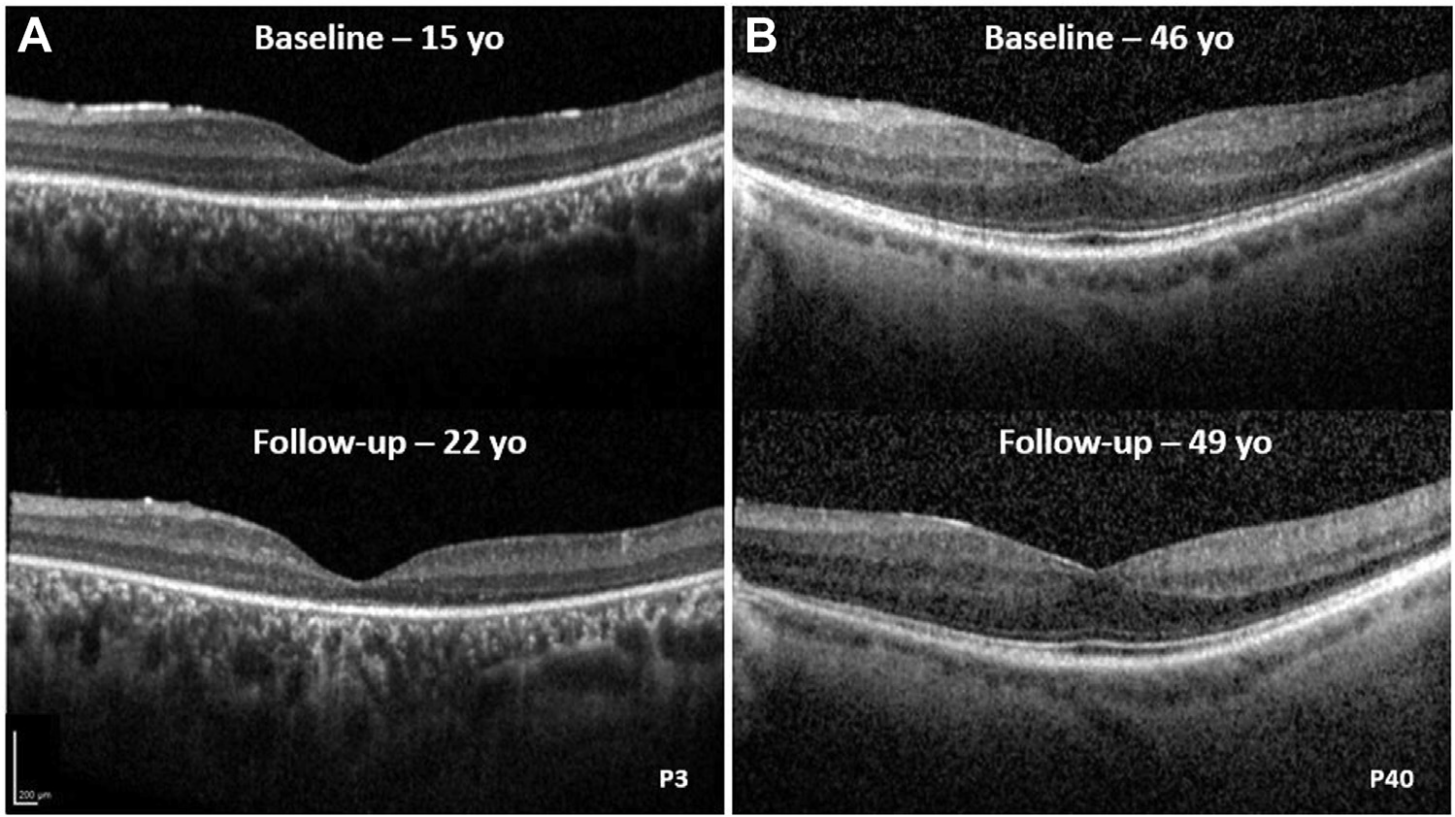
OCT imaging. OCT imaging of 2 patients with RP2-associated retinopathy with (**A**) childhood-onset disease and (**B**) adulthood-onset disease. **A**, Patient shows progressive loss of the ellipsoid zone (EZ) over a follow-up of 7 years, with no identifiable EZ by the age of 22 years. **B**, Patient had a well-preserved EZ at age 46 years, and no EZ loss was observed over 3 years of follow-up. EZW = ellipsoid zone width; ONL = outer nuclear layer.

The 4 patients with adulthood-onset disease (mean age, range: 36.6, 20.5–68.2 years) had evidence of a relatively preserved EZ (mean width 3255 μm, range 615–6500 μm) and ONL (mean width: 89.63 μm, range: 64–129 μm). Three of the 4 patients had longitudinal assessment after a mean follow-up of 3.9 years. The 1 patient with age of onset 17 years progressed to complete EZ loss over 7.4 years (age at follow-up: 31 years). The 2 patients with later-onset disease had stable imaging after 1.4 and 2.9 years (Fig 10).

## Discussion

This study details the clinical phenotype in the largest cohort of genetically characterized patients with *RP2*-associated retinopathy to date, including novel genetic findings. Comprehensive electrophysiological testing, natural history, and serial retinal imaging data highlight the structural and functional spectrum and variability of the disease, with the aim of informing future patient management and interventional trials. *RP2* retinopathy is a predominantly childhood-onset, rapidly progressive retinal degeneration, with macular involvement and early complete loss of EZ in most cases.

In contrast to some other forms of progressive inherited retinal diseases,^39^^,^^40^ there was less dissociation of structure and central vision; central vision was severely decreased in all patients with childhood-onset disease, and the OCT EZ was undetectable by the age of 26 years in most cases. Electrophysiological testing also revealed PERG evidence of macular dysfunction, severe in all but 2 cases. Full-field ERGs were mostly consistent with rod-cone dystrophy, but the severity of generalized (mainly peripheral) retinal dysfunction varied greatly in children and adolescents of a similar age, ranging from undetectable (severe rod and cone photoreceptor dystrophy) to near-normal cone-mediated ERG components (Fig 3).

Rare exceptions of adulthood-onset disease with relative preservation of outer retinal structure (Fig 10) and the wide range of ERG abnormalities in patients of a similar age (Fig 3) highlight the necessity of individual assessments, important to the selection of candidates most suitable for clinical trials and possible future treatment. Patients with complete loss of EZ and geographic atrophy, irrespective of age, are less likely to benefit from attempts to rescue/regain macular function or to arrest progressive maculopathy. There was a rapid rate of progressive EZW reduction and decline in ONL thickness, highlighting a relatively narrow window for intervention, although clinically significant structural changes are likely to be observed within a short time frame in a clinical trial. However, the severity of degeneration may impose challenges in the accurate measurement of such changes.

In the current cohort, we identified 3 patients with a choroideremia-like phenotype similar to some older patients described in a previous study of XLRP,^20^ with advanced degeneration and of older age. It should be noted that none of those patients had a preserved island of vision, and the choriocapillaris atrophy may represent changes secondary to the chronic retinal atrophy. Those cases may highlight the potential value of functional rescue of peripheral retinal function in cases with severe maculopathy, particularly given that some may have near-normal or relatively preserved cone-mediated ERGs (Fig 3).

Ideally, future prospective studies with standardized imaging acquisition protocols need to establish the inter-session repeatability of measurements before being used as outcome measurements in trials. Also, the use of novel high-resolution imaging techniques such as adaptive optics scanning laser ophthalmoscopy may be more sensitive to change.^41^ Prospective natural history studies that monitor patients from a young age will be vital to better establish prognosis, phenotype-genotype correlations, and meaningful end points for trials. Such studies can inform the design of planned treatment trials, including recruitment criteria, assessments, and follow-up time. The preclinical work assessed gene therapy and read-through drugs to make *RP2*-retinopathy an attractive target for intervention.^42^

### Study Limitations

The retrospective nature of the current study has inherent limitations. Follow-up intervals were not standardized, and the functional assessments did not include visual field testing. Further investigation of female carriers who manifest retinal disease will be of value to determine disease severity and inform counseling; moreover, they may also be candidates for intervention.^24^

## Conclusions

This report of a large *RP2*-associated retinal dystrophy cohort helps to define the phenotypic and genetic spectrum. The disorder is characterized by childhood-onset retinal degeneration usually with early macular involvement. Full-field ERGs reveal rod-cone dystrophy in the vast majority, with generalized (peripheral) cone system involvement of widely varying severity in the first 2 decades of life, and OCT imaging shows early complete EZ loss. Novel therapies for *RP2* are under advanced development, and clinical trials are anticipated in the near future. The findings of this study will inform patient management and counseling and are pertinent to the appropriate selection of patients in future clinical trials.
