# Supplementary material for: RP2-Associated X-linked Retinopathy: Clinical Findings, Molecular Genetics, and Natural History
Source: Ophthalmology. 2023 Apr;130(4):413–22. doi: 10.1016/j.ophtha.2022.11.015 (PMC10567581; doi:10.1016/j.ophtha.2022.11.015)
Supplement: Supplementary Methods [file mmc1.pdf]

OCT Assessment

SD-OCT scans were automatically registered to a near infrared reflectance (NIR-R) fundus image. The foveal center is marked on the baseline transfoveal OCT image and the corresponding point marked on the accompanying baseline NIR-R fundus image. The baseline NIR-R overlay is copied and pasted on the follow-up NIR-R fundus image as per vendor software after aligning them. The vertical OCT marker position on the final image is then adjusted to correspond to that shown on the final NIR-R image, so the line scan over the exact same location is identified. This ensures that the same location is selected as the foveal center on both baseline and follow-up OCT images, and is particularly important when locating corresponding positions for follow-up measurements of retinal thickness and the width of the residual ellipsoid zone (EZW).
