# Supplementary material for: RP2-Associated X-linked Retinopathy: Clinical Findings, Molecular Genetics, and Natural History
Source: Ophthalmology. 2023 Apr;130(4):413–22. doi: 10.1016/j.ophtha.2022.11.015 (PMC10567581; doi:10.1016/j.ophtha.2022.11.015)
Supplement: Supplementary_Figure_5 [file mmc2.pdf]

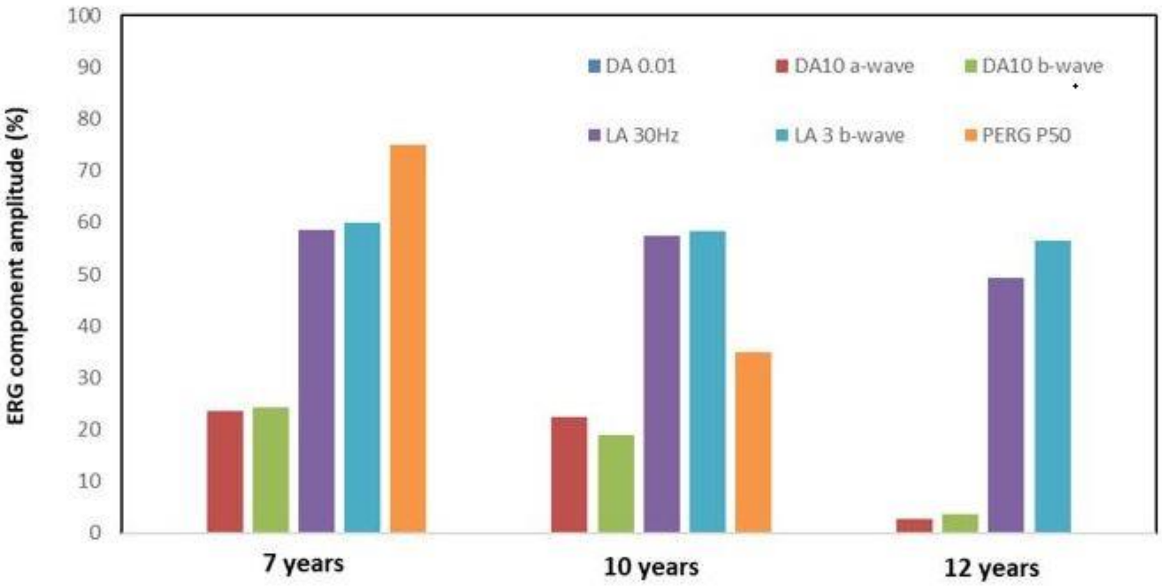

Supplementary Figure 5: Longitudinal Electrophysiology Assessment

Comparison of the main ERG and PERG P50 amplitude parameters at baseline with those obtained at follow-up for patient 21. Ages at the time of testing are shown on the x-axis. The PERG P50 showed progressive worsening and was undetectable at 12 years of age, consistent with progressive macular involvement. The DA0.01 ERG was undetectable on all 3 occasions. The DA 10 ERG a- and b-waves showed marked attenuation from the age of 10 years, in keeping with worsening rod photoreceptor function.
