# Supplementary material for: RP2-Associated X-linked Retinopathy: Clinical Findings, Molecular Genetics, and Natural History
Source: Ophthalmology. 2023 Apr;130(4):413–22. doi: 10.1016/j.ophtha.2022.11.015 (PMC10567581; doi:10.1016/j.ophtha.2022.11.015)
Supplement: Supplementary_Figure_7 [file mmc3.pdf]

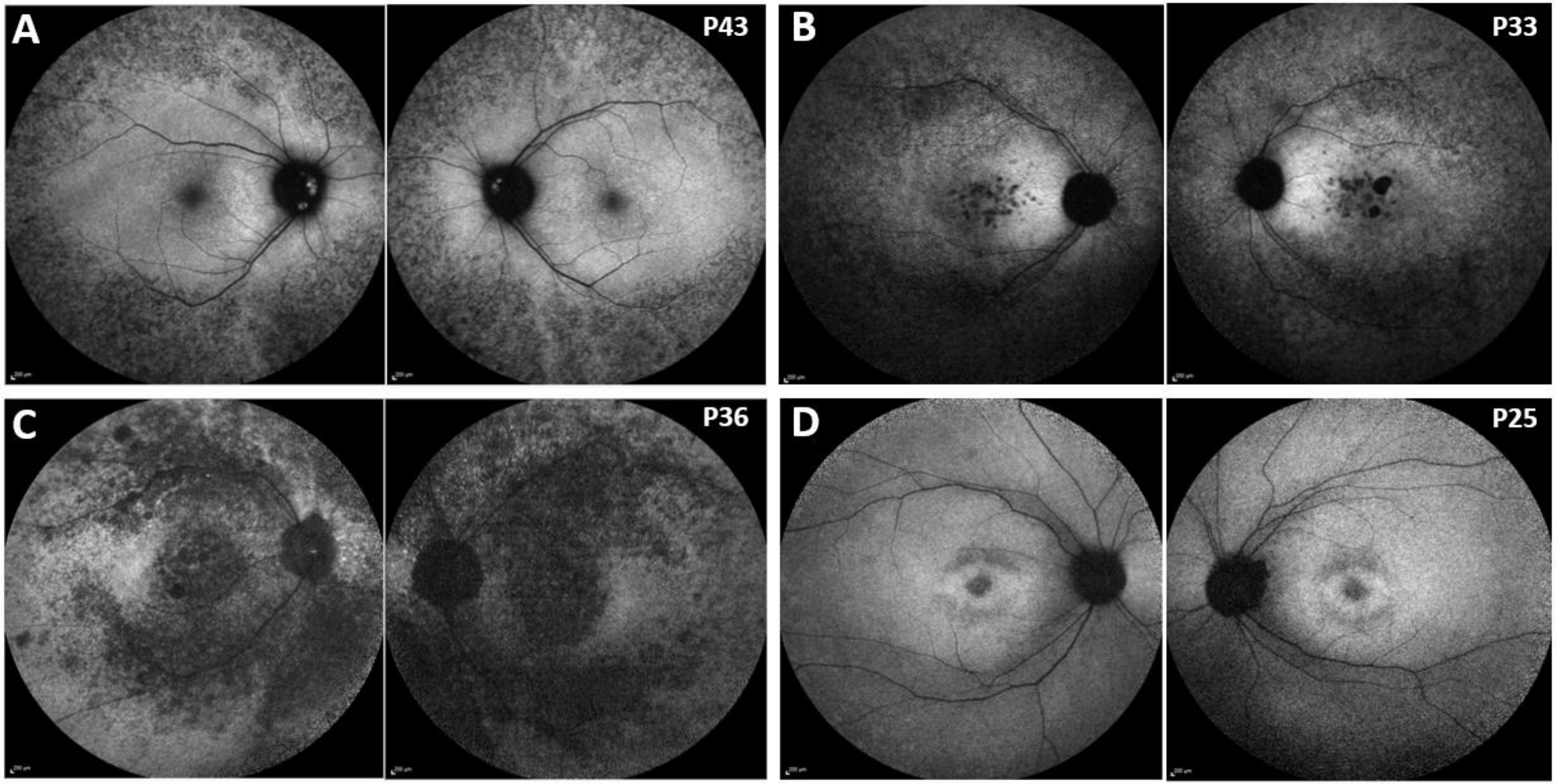

Supplementary Figure 7: Fundus Autofluorescence Imaging Symmetry

Four examples of patients with *RP2*-asociated retinopathy at different stages of the disease. All four patients show a high degree of interocular symmetry of the fundus autofluorescence pattern. (A) Midperipheral patchy signal, with normal foveal pattern at age 20.5 years old. (B) Midperipheral patchy signal, with foveal atrophy at age 24.0 years old. (C) Advancing areas of atrophy in the midperipheral and the fovea at age 27.8 years old. (D) Peri-foveal ring of increased signal at age 11 years old.
