# Supplementary material for: RP2-Associated X-linked Retinopathy: Clinical Findings, Molecular Genetics, and Natural History
Source: Ophthalmology. 2023 Apr;130(4):413–22. doi: 10.1016/j.ophtha.2022.11.015 (PMC10567581; doi:10.1016/j.ophtha.2022.11.015)
Supplement: Supplementary_Figure_9 [file mmc4.pdf]

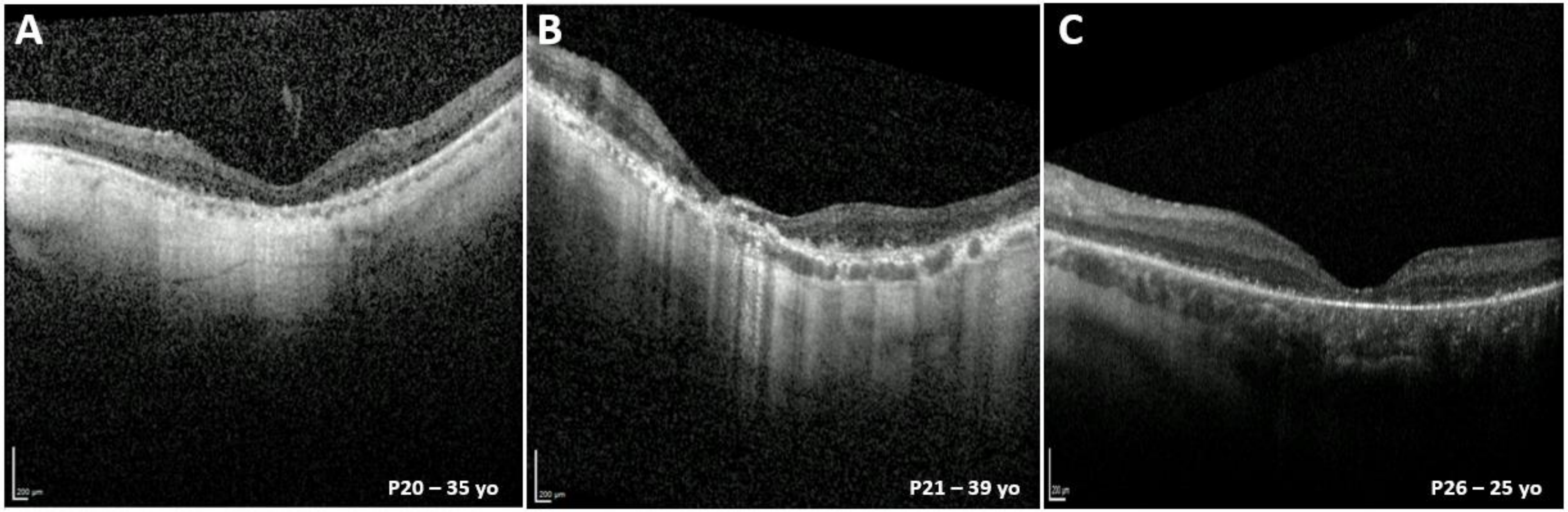

Supplementary Figure 9: Optical Coherence Tomography showing Ellipsoid Zone Loss

Three examples of patients with *RP2*-associated retinopathy with complete ellipsoid zone loss at age (A) 35 years old, (B) 39 years old, and (C) 25 years old. No patient was identified in the current cohort with childhood-onset disease and identifiable ellipsoid zone and age greater than 26 years old.
