# Supplementary material for: RP2-Associated X-linked Retinopathy: Clinical Findings, Molecular Genetics, and Natural History
Source: Ophthalmology. 2023 Apr;130(4):413–22. doi: 10.1016/j.ophtha.2022.11.015 (PMC10567581; doi:10.1016/j.ophtha.2022.11.015)
Supplement: Supplementary_Table_1 [file mmc5.pdf]

Supplementary Table 1: Visual Acuity Grading

**Clinical Severity Grading***	
**Mild Disease**	
BCVA greater than 0.40 LogMAR (20/50)	
**Less severe**	
Late onset (>30 years) and VA worse than 0.40 LogMAR (20/50)	
**Severe**	
BCVA worse than:	at age (years):
0.40 LogMAR (20/50)	<20
0.70 LogMAR (20/100)	21-30
1.00 LogMAR (20/200)	31-40
1.30 LogMAR (20/400)	>41

**WHO Visual Impairment Criteria**		
No or Mild Visual Impairment	≤ 0.48	LogMAR
Moderate Impairment	0.48 -1	LogMAR
Severe Impairment	1-1.3	LogMAR
Blindness	>1.3	LogMAR

*Criteria adjusted from *Jayasundera et al.*
