# Supplementary material for: RP2-Associated X-linked Retinopathy: Clinical Findings, Molecular Genetics, and Natural History
Source: Ophthalmology. 2023 Apr;130(4):413–22. doi: 10.1016/j.ophtha.2022.11.015 (PMC10567581; doi:10.1016/j.ophtha.2022.11.015)
Supplement: Supplementary_Table_2 [file mmc6.pdf]

Supplementary Table 2 : *RP2* genomic variants and predicted effect

cDNA Change	Variant classification	Predicted Protein Change	Exon	Protein Domain	Predicted Effect	Pedigrees (n=38)	Patients (n=54)
c.14_16del	Deletion	p.(Phe5del)	EXON 1	N-terminal acylation	Mislocalization	1	2
c.19A>T	Nonsense	p.(Lys7*)	EXON 1	N-terminal acylation	Loss of Function	1	2
c.43del	Frameshift	p.(Ser15Argfs*31)	EXON 1	β helix domain	Truncation/Loss of Function	2	2
c.128_140del	Frameshift	p.(Ser43Metfs*4)	EXON 2	β helix domain	Truncation/Loss of Function	1	1
c.159_160insAA	Frameshift	p.(Pro54Asnfs*5)	EXON 2	β helix domain	Truncation/Loss of Function	1	1
c.181C>T	Nonsense	p.(Gln61*)	EXON 2	β helix domain	Truncation/Loss of Function	1	1
c.235del	Frameshift	p.(Ala79Leufs*12)	EXON 2	β helix domain	Truncation/Loss of Function	2	2
c.256T>C	Missense	p.(Cys86Arg)	EXON 2	β helix domain	Misfolding/instability	1	1
c.257G>A	Missense	p.(Cys86Tyr)	EXON 2	β helix domain	Misfolding/instability	1	2
c.258T>A	Nonsense	p.(Cys86*)	EXON 2	β helix domain	Loss of Function	1	1
c.338C>A	Missense	p.(Ala113Asp)	EXON 2	β helix domain	Misfolding/instability	1	1
c.341G>A	Missense	p.(Cys114Tyr)	EXON 2	β helix domain	Misfolding/instability	1	3
c.352C>T	Missense	p.(Arg118Cys)	EXON 2	β helix domain	Loss of ARL3 interaction	2	6
c.353 G>A	Missense	p.(Arg118His)	EXON 2	β helix domain	Loss of ARL3 interaction	1	1
c.358C>T	Nonsense	p.(Arg120*)	EXON 2	β helix domain	Loss of Function	5	5
c.450G>A	Nonsense	p.(Trp150*)	EXON 2	β helix domain	Loss of Function	1	1
c.460 G>T	Nonsense	p.(Glu154*)	EXON 2	β helix domain	Loss of Function	2	4
c.568_569delinsG	Frameshift	p.(Pro190Glufs*48)	EXON 2	β helix domain	Truncation/Loss of Function	1	2
c.685_691del	Frameshift	p.(Gln229Alafs*7)	EXON 2	Ferredoxin-like α/β domain	Truncation/Loss of Function	1	2
c.768+1G>A	Splice site		EXON 2/3	Ferredoxin-like α/β domain	Loss of Donor Splice Site	1	1
c.798_801del	Frameshift	p.(Thr267Argfs*5)	EXON 2	Ferredoxin-like α/β domain	Truncation/Loss of Function	1	1
c.884-1G>T	Splice site		EXON 3/4	Ferredoxin-like α/β domain	Loss of Acceptor Splice Site	1	2
c.896C>T	Missense	p.(Ala299Val)	EXON 4	Ferredoxin-like α/β domain	Consequence not known	1	1
c.929dup	Frameshift	p.(Cys311Metfs*18)	EXON 4	Ferredoxin-like α/β domain	Truncation/Loss of Function	1	1
c.969+3A>T	Splice site		EXON 4/5	Ferredoxin-like α/β domain	Loss of Donor Splice Site	1	2
Exon 2 Deletion	Deletion	p.(Val35-Glu256del)	EXON 2	β helix domain	Truncation/Loss of Function	1	1
Exon 5 Deletion	Deletion	p.(Met323-Ile350del)	EXON 5	Ferredoxin-like α/β domain	Truncation/Loss of Function	2	2
Whole Gene Deletion	Deletion	no protein	EXON 1-5	All	Loss of function	2	3
